# Expert insights into public health nutrition challenges: a rapid group model building demonstration workshop

**DOI:** 10.1017/S1368980025100852

**Published:** 2025-08-27

**Authors:** Cindy Needham, Penny Fraser, Carmen Vargas, Laura Alston, Steven Allender

**Affiliations:** 1 Global Centre for Preventive Health and Nutrition, Institute for Health Transformation, Deakin University, Geelong, Australia; 2 Deakin Rural Health, School of Medicine, Deakin University, Warrnambool, Australia; 3 Research Unit, Colac Area Health, Colac, VIC, Australia

**Keywords:** Group model building, Public health nutrition, Community-based systems dynamics, Urban planning

## Abstract

**Objective:**

This paper explored international experts’ views on what influences the development and implementation of local government (LG) planning policy to support healthy food retail environments; and 2) whether a rapid group model building (RGMB) approach can successfully be applied to capture valuable insights.

**Design:**

Adaptation of methods from community-based system dynamics in the form of RGMB.

**Setting:**

In person, facilitated workshop at the World Public Health Nutrition Congress (WPHNC) June 2024, London, England.

**Participants:**

WPHNC delegates.

**Results:**

Sixty-six participants contributed to the RGMB. Factors identified that influence the development and implementation of LG planning to support healthy food retail environments centred around community, evidence, policy, political leadership/priorities and capitalism. Feedback loops identified in the causal loop diagram showed the potential influence of policies to support healthy food retail environments on public health outcomes. Research evidence and data were key factors in supporting community demand for healthy food policies and raising the profile of health as a priority, which, in turn, could support funding to support healthy food environments. Political will and corporate influence in the system were shown to be highly influential.

**Conclusions:**

International experts identified that data is urgently needed to support demand for healthy food policies and political will to address key nutrition issues. The influence of corporate interests was viewed as highly influential over the current system across the world. RGMB workshop activities and processes described in this paper can be used successfully to capture expert insights into complex problems using interactive technologies.

Community-based system dynamics (CBSD) utilises participatory approaches for researchers and practitioners (facilitators) to engage with communities to capture perspectives and address complex problems^(1)^. Group model building (GMB)^(2)^ is a CBSD method that involves tightly scripted and rigorously tested participatory activities to explore how relationships of cause and effect interact to create problems, which are represented in a causal loop diagram (CLD). The CLD provides a visual model that can be used to help participants understand causal relationships, identify leverage points and prioritise solutions to solve complex problems^(3,4)^.

The use of systems thinking to address complex community and social issues is steadily increasing, facilitated by various strategies^(2)^. One significant approach is CBSD, which builds upon socioecological models by emphasising the relationships and feedback loops among different actors, factors, sectors and levels related to a problem. This perspective allows for a visualisation of complicated issues and their potential resolutions in a non-linear way. GMB is a crucial process in CBSD as it facilitates collective actions and deeper insights into a mutually recognised problem through stakeholder involvement^(5)^.

GMB is a method that commonly relies on scripts with rigorously tested, participatory activities to explore how relationships of cause and effect interact to create problems^(3)^. GMB plays a pivotal role in participatory systems thinking, as it enables stakeholders to construct a CLD that visually represents the group’s collective understanding of the issue^(2)^. It provides a visual model that can be used to help participants understand causal relationships, identify leverage points and prioritise solutions to solve complex problems^(6)^.

The GMB approach often involves multiple workshops, each involving structured components and activities including the development of a problem statement, evidence briefs, reference modes (e.g. behaviour over time graphs), connection circles and action ideas^(1,3,6)^. It is acknowledged that there are many different approaches to delivering GMB, and this can vary dependent on time availability, geographic location, demographic of interest-holders and project aims^(6)^. The following steps describe the typical approach used by the research team^(6)^. Prior to commencing a GMB, a key problem statement is formed to guide the discussion, which is usually targeted to a specific age group, geographical location and problem such as healthy eating^(1,3,6)^. The evidence brief provides participants with a brief overview of what we know about the ‘problem statement’ (e.g. whether children in rural areas are eating a healthy diet). ‘Behaviours overtime graphs’ involve asking participants to think about factors (or drivers) that influence the problem in a dynamic way, which are then entered into a connection circle where participants consider how each factor influences another (e.g. increased access to unhealthy food drives consumption of unhealthy food)^(1,3,6)^. The final activity involves participants generating action ideas that have the potential to positively influence important leverage points in the system identified throughout the GMB procedure (e.g. increasing access to healthy food in the local community)^(1,3,6)^.

These approaches provide the potential to gather unique insights where stakeholders are engaged in problems such as digital healthcare^(7)^, child health^(8)^ and nutrition^(9,10)^. While GMB is often facilitated in-person^(11,12)^ challenges like geographic isolation of participants, government restrictions on movement or public meetings (i.e. due to the COVID-19 pandemic), time availability of participants and age have led to adaptations to delivery (such as via online platforms), which allows for exploration of the causes and potential solutions to complex problems^(6,13)^. GMB^(14)^ is typically resource-intensive, and future directions include ways to reduce the time burden and engage high levels of expertise. Opportunities like large conferences, where stakeholders with diverse expertise have gathered, present the chance to simultaneously engage an audience that would be impossible to convene in any other way. One possible implication of this approach is the potentially enhanced credibility of findings due to the inclusion of a more comprehensive perspective gathered from experts from different regions and disciplines providing for greater generalisability of outputs.

International recommendations for local governments (LG) emphasise the critical need for stakeholder engagement and system thinking to implement policy to support healthier food retail environments^(15,16)^. Efforts have been made to understand the successes, barriers and facilitators of implementing policy to support healthier food environments in Australia and internationally^(9,17,18)^ and through review of global documents by the International Network for Food and Obesity/NCD Research, Monitoring and Action Support (INFORMAS)^(19)^. This previous research highlights the challenges to progressing these recommendations experienced by political leadership in the face of strong opposition to these policies and powerful commercial interests^(19)^. However, international expert views on what influences the development and implementation of LG planning policy to support healthy food retail environments have not been captured in the literature. Learnings from public health nutrition practitioners, academics, policy makers and urban planners in other countries, and strengthened international collaboration and consensus on future leverage points could help progress towards^(20)^ achieving healthier food environments.

The 2024 World Public Health Nutrition Congress (WPHNC) brought together over 600 people from more than sixty-six countries committed to public health nutrition, including academics, field workers, students, activists, policy makers and professionals at all levels. Attendees represented a diverse range of perspectives at all levels with the meeting goal of promoting equitable and sustainable access to adequate and nourishing food globally and locally. The broad purpose of the WPHNC was to facilitate in-depth discussions on global nutrition challenges and strategies to address them. During the congress, we introduced and demonstrated the GMB process while asking the participants at the WPHNC:

*What influences the development and implementation of local government (LG) planning policy to support healthy food retail environments?*

The aim of this paper is:

- To explore what international experts think influences the development and implementation of LG planning policy to support healthy food retail environments; and,
- Explore whether a rapid GMB (RGMB) process in an interactive format can successfully be applied in the context of a congress to capture valuable insights.

## Methods

### Setting

In person, facilitated workshop at the WPHNC June 2024, London, England.

### Participants

Participants were registered attendees of the WPHNC that attended the workshop titled ‘Capturing and prioritising the factors that influence healthy food policy: Systems Thinking’. Participants were presented with abstracts for each workshop being held and required to register via online platform to attend. The full abstract can be found in online supplementary material, Supplemental Figure 1. In brief, the abstract outlined that the workshop would build participants capacity and understanding in systems thinking and the GMB process and use this participatory approach to take a deep dive into understanding the broad range of factors that influence the development and implementation of planning policies to support healthy food environments through participating in an RGMB exercise.

The facilitation team included four researchers comprising one key facilitator, two systems modellers and one interim facilitator who provided systems science insights and discussion whilst the systems model was being built.

### Procedure

**Figure 1:**
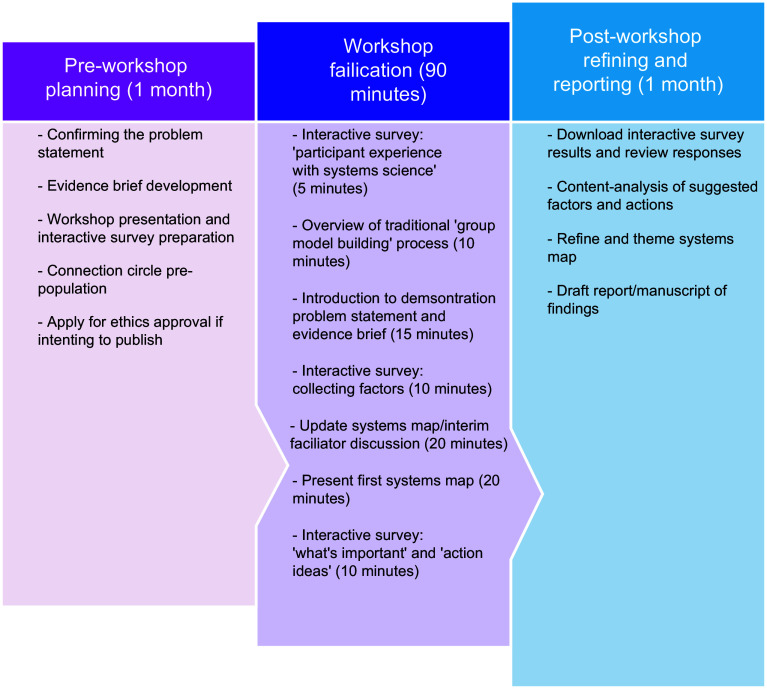
Procedure for planning, facilitation, refining and reporting findings of a Rapid Group Model Building demonstration workshop.

There are three phases in the RGMP process which incorporate adapted activities and scripts from structured GMB workshops^(3)^: (1) pre-workshop planning, (2) workshop facilitation and (3) post-workshop refining and reporting. Activities undertaken in each phase are outlined and presented in Figure 1.

#### Phase 1 – Pre-workshop planning

In the first phase of planning for the workshop, experienced GMB facilitators and public health nutrition researchers (CN, CV and PF) shaped the problem statement for the RGMB workshop. Following consideration of several questions, the problem statement was confirmed to be:

*What influences the development and implementation of local government (LG) planning policy to support healthy food retail environments (i.e. access to healthy food retail outlets and limited unhealthy food retail outlets) in communities?*

Next, a presentation (including an interactive survey and evidence brief) was built in Mentimeter^(21)^. Mentimeter is a platform that allows the creation of presentations with embedded surveys that can be answered anonymously using a mobile phone. In this workshop, participants were asked, ‘how much do you know about systems thinking?’ and ‘have you ever been involved in a GMB workshop before?’. The purpose of this was for the facilitation team to get a sense of the level of understanding and experience of participants in the room in using CBSD. This was followed by an overview of CBSD, including the traditional GMB workshop process and evidence brief. Documents of global relevance were used to provide an overview of global trends in diet, nutrition and prevention of chronic diseases^(22)^, the influential global and local drivers of the obesity pandemic^(23)^, and recommendations for prevention and control of non-communicable diseases^(24)^, followed by an example of progress in healthy urban planning from England^(25)^.

The research team then built an initial ‘connection circle’ using Systems Thinking in Community Knowledge Exchange (STICKE) software^(26)^ guided by relevant research and reports^(9,15–18,23,27–33)^, as well as their relevant expertise in the field. Having a pre-populated connection circle was a time-saving activity, allowing modellers with the opportunity to edit, add or remove factors and connections during the workshop instead of starting from scratch. STICKE software allows for identified causal factors regarding the problem statement to be placed around a circle and then connections demonstrating cause and effect to be identified and agreed upon. STICKE represents these relationships as positive relationships (each factor changes in the same direction – as one factor increases, this leads to the other factor increasing or vice versa) or negative relationships (as one increases the other decreases and vice versa). For example, key factors in implementing healthy food retail policy might include ‘healthy food policy as a priority for government’, ‘corporate power’ and ‘community demand for healthy food’, while connections between them might include an increase in ‘community demand for healthy food’ leading to an increase in ‘healthy food policy as a priority for government’, increasing ‘corporate power’ decreases ‘healthy food policy as a priority for governments’.

#### Phase 2 – Workshop facilitation

At commencement of the RGMB participants were informed that an ethics exemption had been granted for the use of the outputs of the workshop in research, that participation was voluntary, and they were able to attend the session without participating in the interactive surveys which were anonymous. Participants were then given an overview of the GMB process (including reference modes, evidence briefs and graphs over time). The participants were presented with an evidence brief and then asked to consider the problem statement question:

*‘What are the factors that influence the development and implementation of local government (LG) planning policies that support healthy food retail environments (i.e. access to healthy food retail outlets and limited unhealthy food retail outlets) in communities?’*

Participants were asked three interrelated questions with the aim of prompting participants to think of long causal chains or upstream drivers. The first question was ‘w*hat are the factors you think limit the development & implementation of local government planning that supports healthy food retail environments*? The second and third question was *‘thinking of the factor you just shared, what do you think influences it’*? Participants were able to enter answers to each questions as an open-ended answers via Mentimeter^(21)^. Each answer was considered by the research team as a factor (i.e. variables or drivers that influence the problem statement) that were connected (i.e. had an influence on each other).

The two systems modellers considered the input from each participant against the prepopulated connection circle as the responses were being projected on the screen in the workshop. Participants’ responses were then downloaded into an Excel spreadsheet^(34)^, which presented each factor input per participant in individual rows. It was interpreted that the third answer given by participants had direct influence on the second answer which in turn had a direct influence on the first answer. The systems modellers and the key facilitator then spent 20 minutes outside the room and considered each participants’ responses progressively against the prepopulated connection circle. Through discussion by the research team, consensus was reached as to whether the factor was already captured in a similarly worded factor (i.e. no change required), whether an existing factor name needed revision, or a new factor was to be added to the connection circle. The order of each factor suggested by each participant was then considered alongside the existing connections in the circle, with connections removed, added or revised following discussion and consensus being reached by the three researchers. The polarity of connections was determined by the Research Team based on the wording and the positive or negative connotations of the factors submitted by participants. This was assessed in relation to the preceding and subsequent factors, as well as through reflection on the overall problem statement. Consensus was reached through discussion and applied to the CLD. Online supplementary material, Supplemental File 2 presents a sample of responses and how they were interpreted and added to the CLD.

During the 20 minutes the systems modellers and the key facilitator were outside the room, the interim facilitator presented information to participants on how to interpret a CLD. The key facilitator then returned to the room and translated the connection circle into a CLD using STICKE^(26)^, showing a map of all the factors and their connections identified by participants. Several key stories in the CLD, represented by feedback loops and cause and effect structures, were examined and participants were asked to consider ‘*What factors on the map do you think are most important for future action?*’. This was captured via Mentimeter^(21)^ as a word cloud. Participants were then asked, *‘If you could suggest an action idea that would influence positive change in the system, what would it be?’*. Responses to this question were captured via the Mentimeter^(21)^ as an open-ended answer.

#### Phase 3 - Post-workshop refining and reporting

Post-workshop, the CLD was further refined following additional analysis of the suggested factors and in reflection on in-workshop discussions by the lead researcher (CN). A second researcher (PF) then reviewed the CLD to validate the changes made, with consensus reached through discussion as to the final CLD and key feedback loops for presentation in results. Word frequency analysis was undertaken by one researcher (CN) to deductively identify, analyse and report themes within data captured^(35)^. The themes were presented to the research team for review, with consensus reached through discussion.

### Measures of success

To evaluate whether the RGMB interactive format was successfully applied in the context of a congress to capture valuable insights, we utilised the RE-AIM (Reach Effectiveness Adoption Implementation and Maintenance) framework^(36)^. Measures included the number of participants that engaged with the interactive format (reach) and whether the insights captured could be utilised to build a CLD demonstrating insights into the complex issue (effectiveness).

## Results

Sixty-six participants logged into the interactive survey during the workshop.

### Building the causal loop diagram

In response to the question, ‘*What are the factors that influence the development and implementation of local government (LG) planning policies that support healthy food retail environments (i.e. access to healthy food retail outlets and limited unhealthy food retail outlets in communities?*’, fifty-three participants suggested ninety-seven different factors. When asked a second time ‘thinking of the factor you just shared, what do you think influences it’, forty-seven participants suggested a further eighty-two factors that influenced the initial set of factors identified. When asked a third time, thirty-six participants suggested another fifty-three factors. In total, the CLD developed in the workshop considered the 232 suggested factors and their relationships to one another. Through discussion and consensus being reached by the three researchers during the workshop, the 232 suggested factors were consolidated into thirty factors, which were included in the CLD presented in the workshop (see online supplementary material, Supplemental File 3). The full list of factors submitted by participants can be found in Supplementary Table 4.

### Post-workshop refinement and analysis of the causal loop diagram

**Figure 2:**
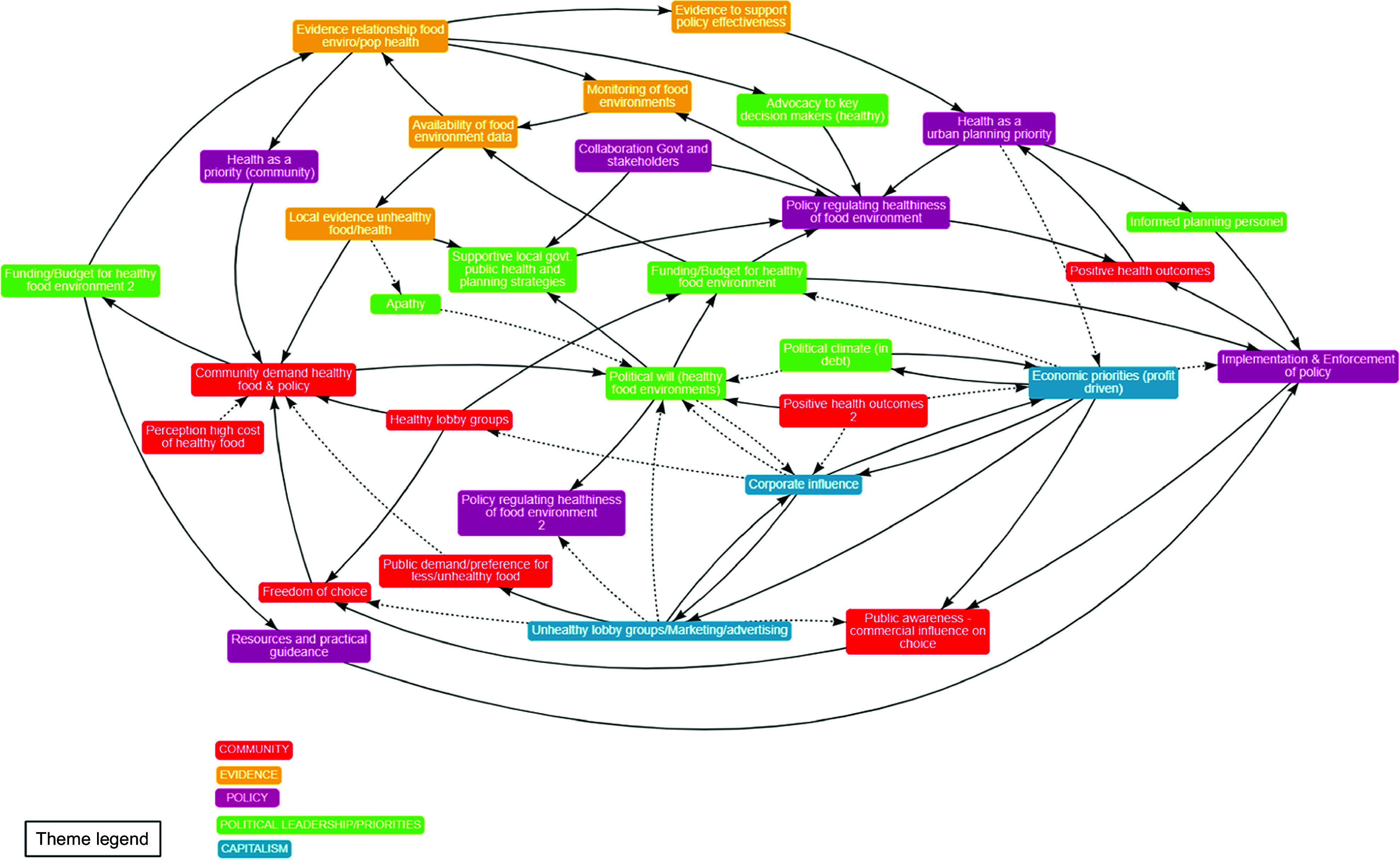
Refined Causal Loop Diagram post Rapid Group Model Building Demonstration workshop presenting the factors that influence the development and implementation of local government (LG) policy to support healthy food retail environments. *Note:* Arrows indicate the direction of influence with a solid line indicating an increase in one factor results in and increase in the other; or a decrease in one factor leads to a decrease in the other. Dotted lines indicate that an increase in one factor results in a decrease in the other; or a decrease in one factor results in an increase in the other.

The word frequency analysis of the suggested factors (see online supplementary material, Supplemental Table 4) showed that the term ‘corporate’ was used most often (*N* 24) across the suggested factors. This was followed by the term ‘political’, which was listed twenty times, with the term used in relation to ‘political will’, ‘personal interest (political)’, ‘political priorities/vested interests’, ‘corporate political power’, ‘lack of political support’ and ‘political climate’. The term ‘lobbying’ including the term ‘industry lobbying’ and ‘food industry lobby’ was used ten times. ‘Capitalism’ (or capital interest in one instance) was listed as a factor twelve times as was ‘funding’ in relation to ‘lack’ or ‘erosion of’ funding. Other key suggested factors included ‘profit’ and ‘resources’ (referring to human and financial). The final CLD included thirty-one factors and is presented in Figure 2. The factors included in the CLD fit into five broad themes being (1) Community, (2) Evidence, (3) Policy, (4) Political leadership/priorities and (5) Capitalism.

### Feedback loops

**Figure 3:**
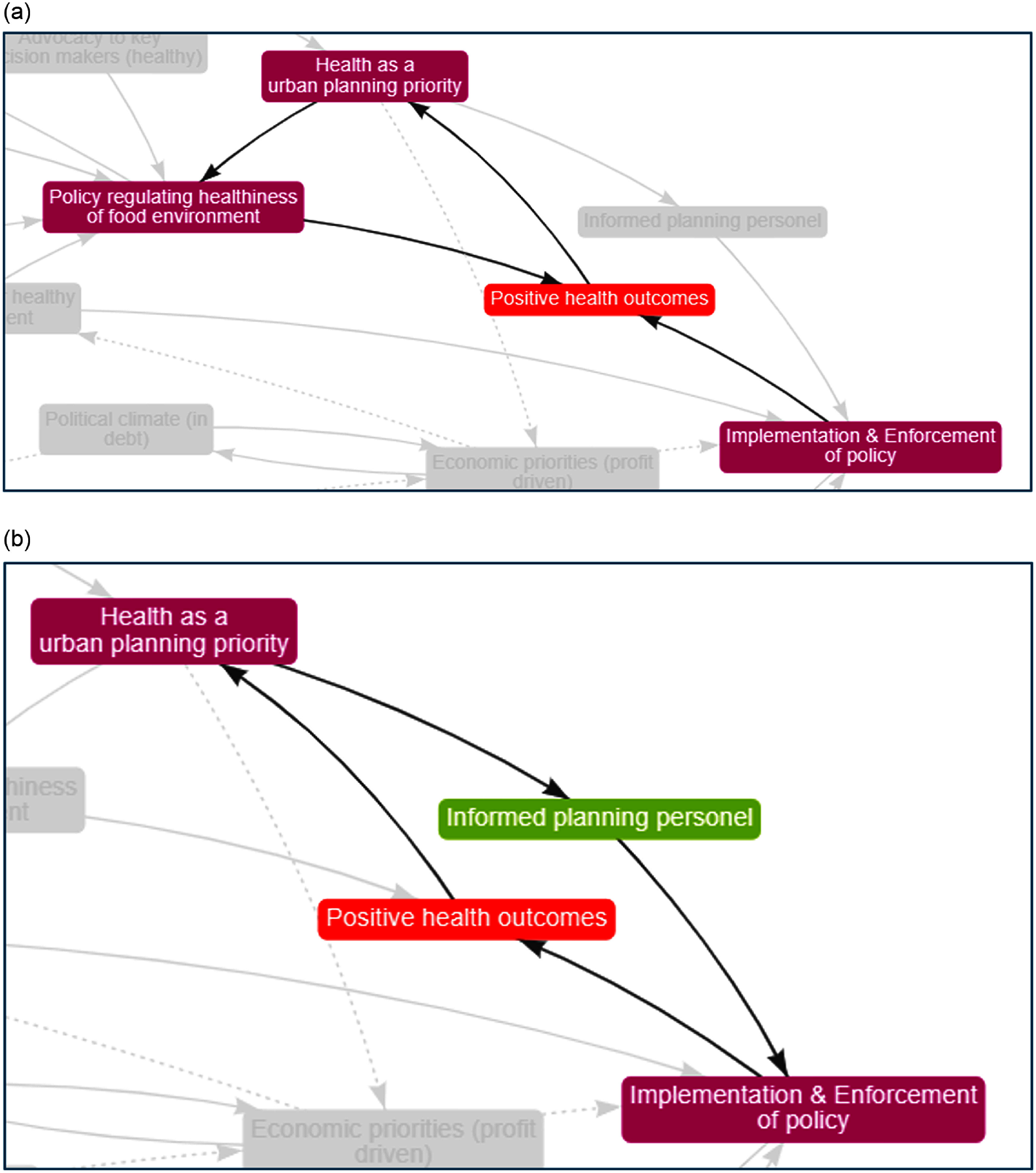
(a) Positive health outcomes build policy momentum. (b) Positive health outcomes raise awareness and motivate implementation and enforcement. *Note:* Arrows indicate the direction of influence with a solid line indicating an increase in one factor results in an increase in the other or a decrease in one factor leads to a decrease in the other.

Five key feedback loops were identified. The first reinforcing loop is centred around ‘positive health outcomes’. Participants described how an increase in ‘health as a urban planning priority’ leads to an increase in ‘policy regulating healthiness of food environment’ resulting in an increase in ‘positive health outcomes’ further reinforcing ‘health as an urban planning priority’ (Figure 3(a)). A second reinforcing loop (Figure 3(b)) shows that an increase in ‘health as a planning priority’ leads to more ‘informed planning personnel’ leading to an increase in ‘implementation and enforcement of policy’ which in turn reinforces ‘positive health outcomes’.

**Figure 4:**
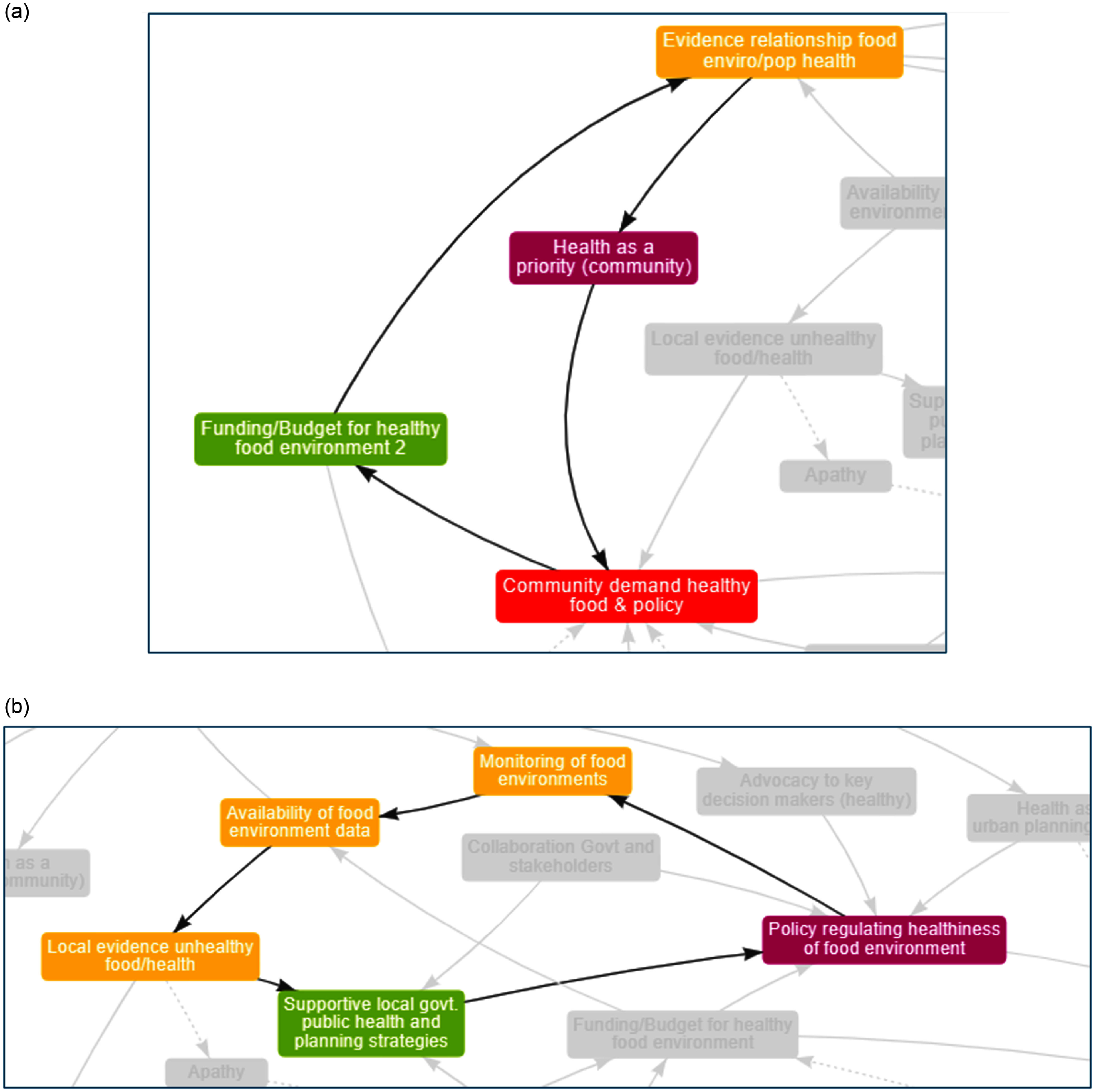
(a) Increased evidence on the relationship between the food environment and health, prioritises health and influences funding. (b) Monitoring of food environments provides local evidence to support policy. *Note:* Arrows indicate the direction of influence with a solid line indicating an increase in one factor results in an increase in the other or a decrease in one factor leads to a decrease in the other.

A further two reinforcing loops centred around factors that related to the theme ‘evidence’. Figure 4(a) shows a reinforcing loop with an increase in ‘evidence on the relationship between the food environment and population health’ leading to an increase in ‘health as a priority’ in the community, thus creating an increase in ‘community demand for healthy food and policy’ influencing an increase in ‘funding/budget for healthy food environments’ further reinforcing and increase in ‘evidence on the relationship between the food environment and population health’. Figure 4(b) shows how an increase in ‘monitoring of food environments’ would lead to an increase in ‘availability of food environment data’ supporting an increase in ‘local evidence unhealthy food/health’; this evidence increasing ‘supportive local government public health and planning strategies’ which leads to increases in ‘policy regulating healthiness of food environments’ which in turn would reinforce ‘monitoring of food environments’.

**Figure 5:**
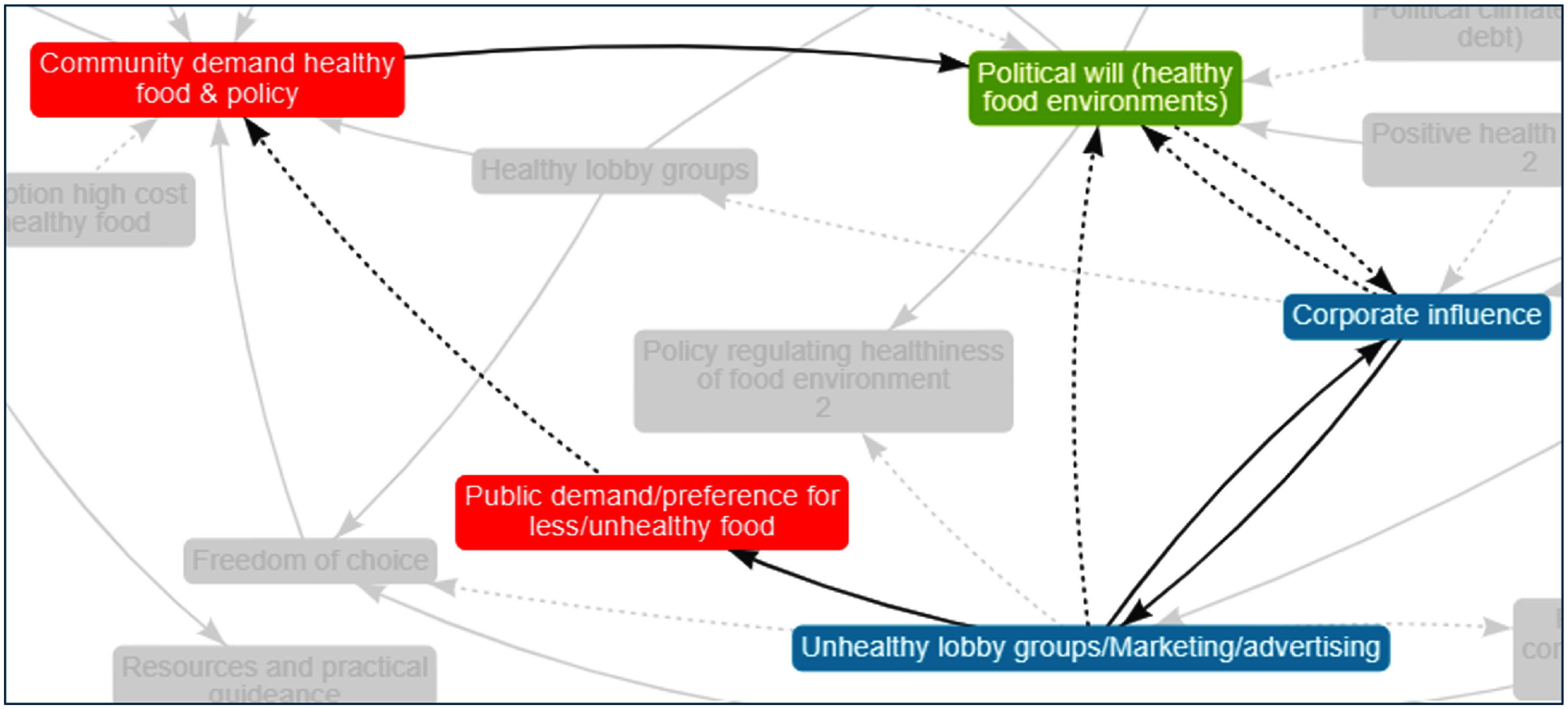
Political will influencing corporate power and community demand. *Note:* Arrows indicate the direction of influence with a solid line indicating an increase in one factor results in an increase in the other or a decrease in one factor leads to a decrease in the other. Dotted lines indicate that an increase in one factor results in a decrease in the other or a decrease in one factor results in an increase in the other.

Figure 5 shows another two balancing loops that centre around ‘political will (healthy food environments)’. Here, we see an increase in ‘political will’ leading to a decrease in ‘corporate influence’ leading to a decrease in ‘unhealthy lobby groups/marketing/advertising’ resulting in an increase in ‘Political will (healthy food environments)’. Being a reinforcing loop, this story could also be true in the opposite direction of influence. For example, a decrease in ‘political will’ may cause an increase in ‘corporate influence’ leading to an increase in ‘unhealthy lobby groups/marketing/advertising’ resulting in further decreases in ‘Political will (healthy food environments)’. There are also two reinforcing loops in this figure. A reinforcing loop exists between ‘corporate influence’ and ‘unhealthy lobby groups/marketing/advertising’ where an increase in one results in an increase in the other (and vice versa) across both factors. A balancing loop can be seen between ‘corporate influence’ and ‘political will’ where an increase in one influences a decrease in the other (and vice versa) across both factors. To the left of the figure, we can see how an increase (or decrease) in ‘unhealthy lobby groups/marketing/advertising’ also feeds into a balancing loop increasing ‘public demand/preference for less/unhealthy food’ which leads to a decrease in ‘community demand for healthy food and policy’ with this leading to a decrease in ‘political will (healthy food environments)’ following through into the loop on the right.

### Where should action take place in the system?

When asked, ‘*What factors on the map do you think are most important for future action*?’ participants identified forty-one factors (see online supplementary material, Supplemental Table 5). Fourteen participants indicated factors related to the theme of ‘policy’, with the most suggested factor being ‘health as a priority’. Eleven participants indicated factors included under the theme ‘political leadership/priorities’ with the most common factor relating to ‘strong leadership’. Of the seven factors that sat within the theme of ‘capitalism’ five cited ‘corporate power’. In the ‘community’ theme, five factors were suggested with ‘community support’ being most common. Three factors were selected within the theme of ‘evidence’ which were all different in wording but largely related to the availability of food environment data.

### What would action to change the system look like?

When asked ‘If you could suggest an “action idea” that would influence positive change in the system what would it be?’ participants suggested thirty-six potential solutions (see online supplementary material, Supplemental Table 6). Empowering and engaging the community to demand and make change was proposed by eight participants. Seven action ideas indicated positive change needed *government* to lead and place health higher on the political agenda. Six actions proposed action that related to *policy and or legislation* around planning powers, food policy, regulation of healthy food retail, taxes on sugar-sweetened beverages and oversight of corporate lobbying groups. Five action ideas proposed *advocacy* activities to raise health as a priority; and *collaboration* by researchers and across sectors was proposed four times. Three actions suggested *regulation* in the form of marketing and promotion restrictions and regulation of unhealthy lobby groups. Two actions focused on research and included continued publishing of evidence to support positive change and funding support. One action idea suggested *behaviour change interventions.*

### Evaluation – reach and effectiveness

A total of sixty-six people (each workshop room had a capacity of 80 + people and the online registration for this workshop was exhausted, albeit exact attendance numbers were not established) interacted with the Mentimeter throughout the RGMB activities, providing a total of 232 potential factors that were considered when building the CLD. When compared to the average participation rate in an online survey of 41 %, our participation rate of 82·5 % and its highest point and 45 % at its lowest reflects an above average participation (reach) in this non-mandatory activity^(37)^. The research team considered the successful consolidation of factors collected from participants into a CLD with five key feedback loops and five themes as an indicator of the effectiveness of the RGMB process in capturing the key factors and relationships reinforcing the nuances of the complex issue.

## Discussion

This is the first paper to use RGMB to outline international perspectives on ‘What influences the development and implementation of LG planning policy to support healthy food retail environments’. The RGMB workshop provided 386 points of data input (i.e. the number of individual responses to proposed questions throughout the RGMB) by sixty-six public health experts from around the world in 90 minutes. Results showed that a RGMB workshop can be a valuable method of both demonstrating GMB process and capturing insights into complex issues. The key themes demonstrated in the CLD suggest the factors that limited the development and implementation of LG planning that supports healthy food retail environments are centred around community, evidence, policy, political leadership/priorities and capitalism. Feedback loops identified in the refined CLD showed the potential influence of policies to support healthy food retail environments on public health outcomes. Research evidence and data were also seen as key factors in supporting community demand for healthy food policies and raising the profile of health as a priority, which, in turn, could support funding to improve the healthiness of food environments. Political will for healthy food environments and corporate influence in the system also were shown to be particularly influential. Factors participants indicated were important for future action included government action, policy and/or legislation and advocacy.

The CLD and the stories within resonate strongly within frameworks and strategies from international^(16,19,38)^ and national^(39–41)^ governments and organisations which aim to prevent non-communicable diseases and the increasing prevalence of people with obesity through ensuring supportive healthy food environments. Furthermore, outputs strongly reflect the key factors and potential leverage points for change identified in one Dutch municipality with a smaller (*n* 18) participation group through two structured GMB workshops exploring a similar topic^(42)^. In this study, the leverage points and systems-based actions to foster healthy and sustainable local food systems identified forty-six factors shaping the local food environment and eight leverage points including societal, individual socio-economic, commercial, government policies and political factors^(42)^.

The focus on factors that relate to capitalism within the CLD indicates that addressing the commercial determinants of health^(43)^ will be a key factor in the development and implementation of LG planning that supports healthy food retail environments. The collective term ‘policy inertia’ reflects well the stories being told in the CLD, suggesting that strong opposition from commercial stakeholders that seek to benefit commercially from unhealthy dietary habits was slowing progress for the development and implementation of healthy food environment policies by political leaders^(19)^. Our data shows the similarities in views of this system globally, and supports a need for international experts to collaborate and share lessons learned to support improvements in LG action for healthier food environments globally. This could be achieved through more consistent use of GMB to build global consensus and subsequent action, given the power dynamics of commercial and corporate influences. Such powerful influences can be better addressed locally through support and knowledge sharing at an international level.

Emphasising the critical importance of government action to increase the healthiness of the food environment, the Government Healthy Food Environment Policy Index (Food-EPI) was developed, setting standards for good practice in improving food environments and implementing obesity and non-communicable disease prevention policies and actions^(16)^. The framework was developed following a review of policy documents on reducing obesity and non-communicable diseases from international agencies, national government agencies, global non-government agencies, professional societies and expert advisory groups^(16)^. Within the framework there are two domains under which the key components are classified which are *Policies* and *Infrastructure Support* ^(16)^. The key themes in the CLD and proposed action ideas for systems change identified through the RGMB show clear alignment with proposed good practice within each domain^(16)^. In relation to the policies component of the framework, the domains *Food Retail* and *Food Prices* were highlighted for future action, particularly relating to the availability of robust zoning laws and policies to restrict predominantly unhealthy food retailers, as well as fiscal policies that would make healthier food more affordable than unhealthy products (e.g. sugar-sweetened beverages at a higher price)^(16)^. The components of the *Infrastructure and Supports* were also highlighted across a number of domains namely *Leadership, Governance, Monitoring and Intelligance* and *Funding and Resources.* The domain of *Leadership* and *Governance* was probably most emphasised, with findings supporting the proposed good practice of having strong visible political support for improving the healthiness of the food environment and public health nutrition, as well as having robust processes for restricting commercial influences on the development of policies to improve population nutrition that relate to food where conflicts exist ensuring accountability and transparency^(16)^. Food-EPI as of the time of writing (September 2024) is actively being used in fifty-six countries across the globe and can serve as a tool to make governments at all levels accountable for meeting the proposed standards. A report on government Food-EPI progress from Australia shows that while success is being made in some areas to meet the proposed good practice in each domain, for example, the development of the National Obesity Strategy^(39)^, there is still much work needed to meet the desired measures of success across all domains^(32)^. Into the future, the Food-EPI framework has the potential to be used by communities and public health agencies to advocate for action where governments are inactive in meeting the targets, and for researchers to advocate for resources to support monitoring activities^(44)^.

Exploring further with world-leading experts in public health nutrition how we might seek to achieve health as a priority, and not profit, would be beneficial for future success in prevention efforts. Progressing the insights captured in the RGMB demonstration workshop using systems science tools and techniques has the potential to engage the collective for future impact.

### Strengths

This RGMB workshop demonstrates a method to engage participants attending conferences or similar events where international experts are in attendance for short periods. The RGMB workshop demonstrates the GMB process and can be effectively modified to provide systems science education. By incorporating practical activities alongside interactive surveys, an extensive amount of data can be captured. This adaptation not only reduces the resource intensity of conducting a regular GMB (e.g. 3 workshops and 7–10 hours) but also enables a deeper understanding of complex systems from participants with different views and nationalities, ultimately leading to more targeted research that can lead to better-informed decision-making and innovative problem-solving solutions. This allows for a rigorous review of the CLD post-workshop, providing valuable insights into participants’ perspectives on the complex issue.

### Limitations

With the workshop structure and Mentimeter^(21)^ settings, the analysis was limited in some instances. First, we did not limit the number of responses participants were able to submit in relation to the problem statement. As a result, several participants added more than one response in the first line of questioning, reflecting a larger number of responses which decreased with each successive line of questioning. However, like in a workshop setting, this may have been reflective of participants having exhausted the factors they wish to highlight in relation to the issue. In future iterations of RGMB, we would recommend researchers limit to one response per line of questioning. When asked ‘what factors on the map do you think are most important for future action?’ participants were unable to see the CLD at the same time as the interactive screen which resulted in some participants incorrectly entering the factor name which resulted in forty-one unique factors being identified due to variation in wording. Given the international nature of the WPHNC, the results do not account for the contextual differences in local planning issues. Due to time constraints, participants were unable to revise the CLD and provide further feedback; this may have reduced the sense of ownership commonly attributed to this process. In future iterations, finding ways to enable participants to provide feedback after the session could help foster relationships with diverse sectors, enhance problem-solving motivations and lead to a more accurate CLD.

While we were able to capture interesting insights from participants who attended the workshop, the refined CLD only depicts the researcher’s interpretation of the factors proposed. Forgoing the rigorous steps undertaken in the traditional CBSD GMB workshop series limits the validity of the results presented because of the rapid translation of participant input into a smaller refined CLD. A full GMB workshop series typically includes three workshops totalling seven or more hours, and a significant amount of time between workshops by the facilitation team to refine and prepare resources. Extensive notes are taken to allow for further clarification, a step that was not done in the current RGMB. We did not allocate the time to discuss the story behind each factor proposed by participants, nor did we give participants the opportunity to review the model and make corrections that would normally occur. In addition, the action ideas component of a GMB, which usually takes place over two to three hours and involves a series of discussion groups and prioritisation steps, was not undertaken, and the action ideas are presented as proposed only. Finally, it is possible that the responses provided by participants may have been influenced by earlier presentations attended at the conference and by the responses submitted to the interactive survey, which were projected in the room. However, CBSD GMB operates within a social constructionism paradigm, recognising that the experiences and perspectives of both participants and the research team will collaboratively shape the knowledge generated in the final CLD^(45)^. Given the above limitations, it is possible that the researchers may have misinterpreted proposed factors and stories presented within the CLD, and or the purpose of proposed action ideas. As such, all results should be interpreted with caution. Moreover, all these points can be considered when planning and conducting future iterations of RGMB.

### Conclusion

Views from international delegates suggest the factors that limit the development and implementation of LG planning that supports healthy food retail environments are centred around community, evidence, policy, political leadership/priorities and capitalism. The ubiquity of this view across the system globally supports a need for international experts to collaborate and share lessons learned to support improvements in LG action for healthier food environments. Rapid demonstration of GMB workshop activities and processes described in this paper demonstrate that they can be used successfully to capture expert insights into complex problems using interactive technologies. Unique situations where global experts are available at a given time and location and have an interest in learning about CBSD and GMB process provides an opportune scenario for knowledge capture using these participatory techniques.

## Supporting information

Needham et al. supplementary material Needham et al. supplementary material

## Supplementary material

For supplementary material accompanying this paper visit https://doi.org/10.1017/S1368980025100852
