# Supplementary material for: Expert insights into public health nutrition challenges: a rapid group model building demonstration workshop
Source: Public Health Nutr. 2025 Aug 27;28(1):e150. doi: 10.1017/S1368980025100852 (PMC12516629; doi:10.1017/S1368980025100852)
Supplement: Needham et al. supplementary material [file S1368980025100852sup001.docx]

**Supplementary file 1. Workshop Abstract, World Public Health Nutrition Congress 2024**

**Title:** **Capturing and prioritising the factors that influence healthy food policy: Systems Thinking**

Background: System science offers unique methods to understand the relationships between all parts within a system. Group Model Building (GMB) is a recommended method for using system science which involves a series of facilitated workshops through which key stakeholders explore the causes of complex problems and generate potential interventions informed by current evidence. Aims/Learning objectives: Participants will learn how GMB techniques can be used to develop a Causal Loop Diagram of a complex problem such as ‘what are the factors that influence the development and implementation of planning policies that support healthy food environments’. Through this participatory approach participants will take a deep dive into understanding this complex problem and how they interact with each other from the perspective of delegate representatives from around the globe. Process: Participants will be introduced to, and participate in a rapid GMB exercise in which we will build a Causal Loop Diagram (CLD); providing a visual representation of the factors (barriers and facilitators) of the identified complex problem. Active participation will be achieved through the use of online participatory tools in identifying factors, actions already happening and areas where future action should be focused. Capacity building: Through participating in the GMB, participants will build their capacity and understanding in Systems Thinking, the GMB process, and the use of Deakin’s STICKE software to build CLDs. They will also build their understanding of the broad range of factors that influence the development and implementation of planning policies to support healthy food environments.

**Supplementary file 3. Causal loop diagram presented in the Rapid Group Model Building demonstration at the World Public Health Congress 2024, London UK.**


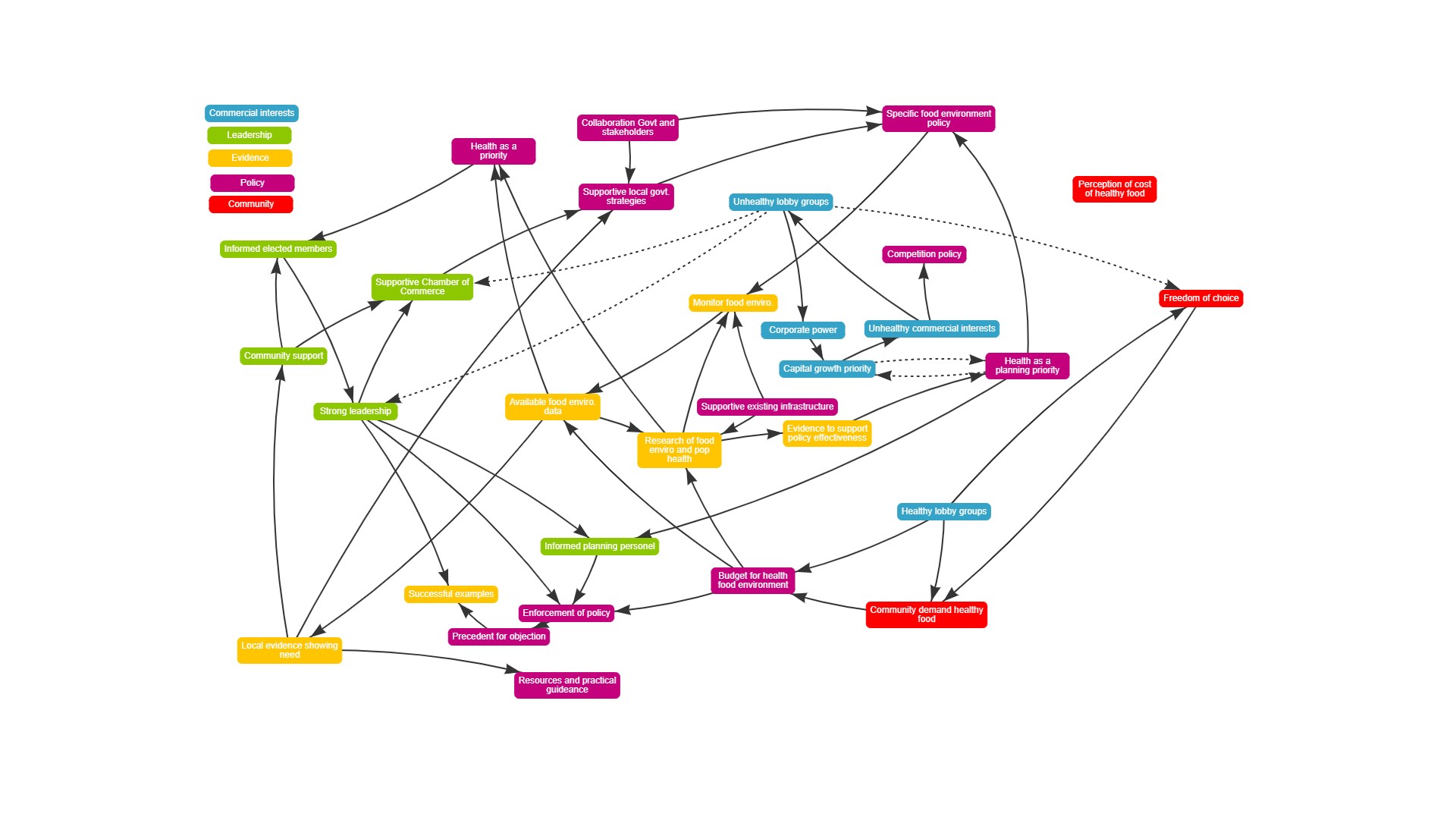


Note: Arrows indicate the direction of influence with a solid line indicating an increase in one factor results in and increase in the other; or a decrease in one factor leads to a decrease in the other. Dotted lines indicate that an increase in one factor results in a decrease in the other; or a decrease in one factor results in an increase in the other.

Supplementary file 4: Submitted factors that influence the development and implementation of local government (LG) planning policies that support healthy food retail environments in workshop

| **Participants** | **What are the factors you think limiting the development & implementation of local government planning that support healthy food retail environments?: 1** | **Thinking of the factor you just shared, what do you think influences it?: 1** | **Thinking of the factor you just shared, what do you think influences it?: 2** |
| --- | --- | --- | --- |
| 1 | Lack of local authority resources; Local authorities prioritising other higher risk issues (local knife crime, food safety etc) | Lack of trained local authority enforcers | No government funding at national level |
| 2 | Corporate power; Health as a low priority | Profit of junk food leads to corporate influence |  |
| 3 | Human and financial resources Clear mandates Intersectoral coordination Other priorities No golden standards | Advocacy; Budget plan; Priority setting; Strengthening competencies; Intersectoral coordination unit | Champions; Media |
| 4 | Corporate lobbying; Conflicting political priorities | Unchecked corporate access to decision makers | Corporate power |
| 5 | Lack of political action | Budget constrains; Fear | Education; Awareness |
| 6 | Cost; Lack of care to improve more deprived areas; Competing priorities | Taxes | Political climate; Lack of consequences |
| 7 | Commercial influence | Neoliberal political paradigm favouring small government /limited government intervention through regulation; Colonialism |  |
| 8 | Unwilling to interfere with industry/businesses for the “economy” | Business/food industry |  |
| 9 | Industry lobby | Market structure | Lack of regulation |
| 10 | Lack of demand from civil population; General population lack of consciousness about the problem (no political revenue) |  | Education and values |
| 11 | Political inertia; Conceptions around freedom of choice | Corporate lobbying; Political identity / polarization | Degradation of public sector governance |
| 12 | Apathy; Short-term economic focus; Belief that individuals can make informed choices; Other priorities | Cost pressures; Lack of resource | Insufficient budget or government investment |
| 13 | Nutrition as personal responsibility; Not considered in rezoning or planning; Small business | Resourcing; Academic silos | Academic silos |
| 14 | Lobbying; Lack of practical know-how; Changing government agendas | Lack of transparency in government systems; Economic climate | Lack of incentives; Unregulated markets |
| 15 | Business | Policies and government |  |
| 16 | Industry interference; Lack of political will; Misunderstanding why this is important | Resources of industry to further their agenda; Public focused on other issues, I.e cost of living pressures | Government inaction to control power of industry |
| 17 | Industry involvement; Competing priorities for budget strapped local authorities; Profit seeking | Too wide of a mandate for Las | Capitalism; Flawed government structures and resulting mandates |
| 18 | Available food environment data |  |  |
| 19 | Power imbalances and diverging (short-term) interests of different stakeholders; Apathy | Personal buy-in from leaders / champions; Time constraints | Too many emergencies; Missing (long-term) coordination and outlook |
| 20 | Food industry lobby | Food industry power and capital | Capitalism; Values of society |
| 21 | Lobby | The capital interest | Capitalism |
| 22 | Food industry (and others related) lobby | Capitalism | Capitalism |
| 23 | Limited multisectoral engagement between departments of health, trade, finance, etc | Poorly designed or implemented multisectoral policy making mechanisms. | Government's administrative capacity |
| 24 | Lack of support/mandates from national government; Lack of coordination/power in civil society | Corporate lobbying; Funding | Political climate |
| 25 | Industry opposition | ***Profit driven industries; Capitalism*** |  |
| 26 | Consumer demand for less healthy foods; Ease, cost, influence: Consumers leading busy lives & seeking less expensive food products & convenience foods; habit; demand from other family members; advertising/marketing |  | Gender imbalance; socioeconomic inequality; corporate power |
| 27 | Private sector; Profit-driven mindset |  | Capitalism |
| 28 | ***Political inertia; Emphasis on industry  self regulation not on governmental regulation; Profit margins*** | Prioritizing economic growth | Capitalism; Capitalism at the cost of public health priorities; |
| 29 | Lack of political will to act; Insufficient criteria defining healthy food retail | Political gridlock prevents action; Unhealthy foods cheaper to produce | The food system is optimized for profit not health; ; |
| 30 | Lack of collaboration across different departments | Siloed thinking | Short term thinking |
| 31 | Industry lobbying/lawsuits; Enforcement budget; Employment provision; Economics of unhealthy food; Diapered government agency/authority; | Government funding/tax; Economics; Employment; Industry budget vs authority budget; Consumer demand | Employment structure; Supply/demand; Government action |
| 32 | commercial interests, perception of costs and benefits | existing governance structures | neoliberalism |
| 33 | Lack of coherence at different levels  Lobbying | Different priorities Economic system - Influence of industry | ***Dependence on funding from the industry Capitalistic system Personal interest (political)*** |
| 34 | Lack of govt interest; Space ( zoning policies are tricky as the country is small) | Lack of understanding, commercial interests , basic ideologies (for example Neo-liberalism thinking) | Values |
| 35 | Economic power of big retail companies | Liberalism. | Capitalism |
| 36 | Funding - lack of. Political wills for  Industry  Capacity f | Bottom line |  |
| 37 | Political priorities/vested interests Resources  Lack of understanding of the importance of this by the policy makers Disconnect between political wants and can community needs | Public support Political will Lack of understanding by policy makers of the importance of these measures for population health; Short-term V's long-term political gain | Territorial thinking  Funding model-funding silos |
| 38 | Lack of evaluative evidence; Concerns re economic impacts on community; Concerns re impact on local retail 'vitality'; Corporate interference; Limited resources in local government to develop innovative policy; Assumption of low public support | Lack of evidence; Fragmentation of effort across multiple local governments; Limited resources in local government; Limited resources in local government | Central government erosion of local government funding; Corporate political power |
| 39 | Unorganized healthy and fresh food retail or lack of business acumen | Education level of business operators; Lack of public awareness |  |
| 40 | Infrastructure and logistics; Competing priorities | Planning and funding allocation; Lack of political support | Responsibility |
| 41 | Lack of action at the national level undermining action at the local level. Local governments not having the power/mandate to intervene | Lack of political will at the national level, low on the national priority agenda |  |
| 42 | Negative impacts if planning was implementer, e.g. empty real estate if jo food outlets | Move to online shopping, empty shops turned into food outlets; Benefits will not be evidenced within the lifetime of a government | Austerity; Influence national drivers Inform elected members Build community movement for change; |
| 43 | Cost, corporate lobbying, competing priorities | People's autonomy over what to eat and not to eat. Consumerism that helps corporates; People's autonomy over what to eat. Increasing consumerism |  |
| 44 | Low political will; Lack of legislative power at local levels; Public don't vote in local elections | Lack of civics education; Misinformation campaigns funded by corporate actors | Conservative politics |
| 45 | Capacity |  |  |
| 46 | Corporate influence; Taxes | Funding; Limited knowledge and awareness |  |
| 47 | Lack of data on consumer eating behaviour and lobby | Lack of financial resources for data collection and gathering; Lack of regulation to tackle lobby from the transnationals | Policy inertia & neoliberalism |
| 48 | Central government | The politics of the Government in power | Political climate |
| 49 | Commercial influence  Diverse sectoral priorities within government; Conflicting sectoral priorities; Low enforce resources | Conflicting governance goals; Global economic paradigms; Trade and investment agreements | Value systems |
| 50 | Missing mandate | Importance of foods / nutrition towards population health underestimated by politics; Industry pressure |  |
| 51 | Commercial interest lack of motivation  No regulations or enforcing policy; Big food preventing regulation; Perceived economic need |  |  |
| 52 | Influence food industry | ***Profit; Capitalism*** |  |
| 53 | Corporate interest; Conflict of interest | Local economic pressure; Spin doors / decision makers who have some interest on local retail | Neoliberalism; Open trade markets; |

**Legend**

| Profit |
| --- |
| Commercial |
| Political |
| Capitalism |
| **Bold = contains two terms listed above** |

**Supplementary file 5. Factors participants identified were the most important on the map for future action?**

| What factors on the map do you think are most important for future action? | Theme |
| --- | --- |
| Budget Strong_leadership Local_evidence_showing_ne | **Political leadership/priorities** |
| Enforcement Leadership Consumer_demand |  |
| Informed_elected_members Community_support Specific_food_environment |  |
| Informed_planning_persone Available_food_env_data Unhealth_commerc_interest |  |
| Political_will Commercial_incentives More_research_needed |  |
| Resources_and_practical Strong_leadership Policy |  |
| Strong_leadership Capital_growth_priority Community_support |  |
| Strong_leadership Community_support |  |
| Strong_leadership Corporate_power |  |
| Strong_leadership Food_environment_research Supportive_local_gov |  |
| Strong_leadership Informed_planning Supportive_local_govt |  |
| Leadership Policy Evidence |  |
| Capital_growth_priority Enforcement_of_policy Health_as_a_priority | **Capitalism** |
| Corporate_power |  |
| Corporate_power Capita_growth_priority Unhealthy_lobby_groups |  |
| corporate_power healthy_lobby_groups budget |  |
| corporate_power monitor_food_environment specific_food_environment |  |
| Corporate_power Unhealthy_lobbying Strong_leadership |  |
| Unhealthy_lobby_groups Food_environment_data Health_lobby_groups |  |
| Community_demand_healthy Research_on_food_environm Budget | **Community** |
| Community_engagement Funding |  |
| Community_support |  |
| Community_support Monitor_food_environment Evidence_of_policy |  |
| Community_support Policy_enforcement Leadership |  |
| Available_food_envir_data Strong_leadership Healthy_lobby_groups | **Evidence** |
| Local_evidence_showing_ne Supportive_govt_strategy |  |
| Measuring_food_environmen Budget Healthy_lobby |  |
| Successful_examples Monitor_food_environments Budget_for_healthy_food_e | **Policy** |
| Collab_with_stakeholders Evidence_of_policy_effect Health_as_a_priority |  |
| Collaboration_govt_and_st |  |
| Competition_policy Available_food_environ Policy_enforcement |  |
| Connection Support |  |
| Health_as_a_priority |  |
| Health_as_a_priority Budget_for_healthy_food_e |  |
| Health_as_a_priority Commercial_incentives Community_support |  |
| Health_as_a_priority Corporate_power Budget_for_health_food_en |  |
| Health_as_a_priority Enforcement_of_policy Healthy_lobby_groups |  |
| Local_l_gov_strategies Health |  |
| policy_coherence commercial_interests Leadership |  |
| Supportive_govt_strategie Strong_leadership Monitoring_food_environ |  |
| Food_environment_policy Enforcement_of_policy community_demand_healthy |  |

Note: Theming is based on the first factor listed.

**Supplementary file 6. Proposed ‘action ideas’ for systems change**

| Theme | If you could suggest on Action Idea that would influence positive change in the system what would it be? |
| --- | --- |
| Advocacy | Advocacy coalitions working with same goals |
| Advocacy | Advocate to political leaders who can make health a priority in policies |
| Advocacy | Change the narrative to healthy diets |
| Advocacy | Fund health advocacy |
| Advocacy | Strengthen healthy lobby groups and make them acvountable |
| Behaviours change | Behaviour change interventions forming neighborhood networks |
| Collaboration across sectors | Breaking down silos |
| Collaboration across sectors | Cooperation at different levels |
| Collaboration across sectors | Establish a leadership network to influence healty food retail policy making |
| Collaboration of researchers | Academic coalition |
| Empower | Encourage and incentivize local communities to congregate, discuss issues and prioritize key action points to establish agendas and start implementing change. |
| Empower | Genuine community engagement |
| Empower | Give the voice to the community |
| Empower | Socialism |
| Empower | Empower civil society |
| Empower | Empower community with knowledge and skills to demand change |
| Empower | Generate demand for healthy food. Mandate unhealthy sellers to sell healthy |
| Empower | Media campaign to build community support |
| Evidence | I think I should focus on publishing to get the evidence out there |
| Funded research | Money on research to ground tackled interventions to promote health |
| Govenrment action | Coordinated action between national and local governments |
| Govenrment action | I think the key action should be in building political consensus for action. Because more science won’t make it to policy without political will, and commercial interests will just have to adapt. |
| Government action | Empowering of local government from state government to build leadership |
| Government action | Put pressure on political contacts to make healthy food a priority |
| Health in Government | Health practitioners encouraged to get into positions of political power! |
| Health in Government | More popular governance |
| Health in Government | Provide time and resource to build local government food policy leadership. |
| Marketing & promotion restrictions | Focus on restrictions on marketing and promotions will the retail environment |
| Policy/Legislation | Mandates from national gov to reduce unhealthy food retail to give power to local planners |
| Policy/Legislation | Policies centered on the right to food |
| Policy/Legislation | Regulation for food retailers |
| Policy/Legislation | Regulatory policies |
| Policy/Legislation | Take into account community needs for higher demand of heatlhier foods and prioritze them thriugh policy (cooking skills, time, preferences, affordability) |
| Policy/Legislation | Tax SSBs to fund interventions |
| Regulation lobby groups | Greater regulation of unhealthy lobby groups |
| Regulation lobby groups | I think the action should be transparency of corporate lobbying and donations which will affect the corporate power and interests part of the system |
